# The impact of demonstration plots on improved agricultural input purchase in Tanzania: Implications for policy and practice

**DOI:** 10.1371/journal.pone.0243896

**Published:** 2021-01-15

**Authors:** Haroon Sseguya, Daniel S. Robinson, Hamisi R. Mwango, James A. Flock, Julius Manda, Rodrigo Abed, Silvanus O. Mruma

**Affiliations:** 1 International Institute of Tropical Agriculture (IITA), Regional Hub for Eastern Africa, Dar es Salaam, Tanzania; 2 U.S. Agency for International Development, Bureau for Resilience and Food Security, Washington, DC, United States of America; 3 ACDI/VOCA, Feed the Future Tanzania NAFAKA II Activity, Iringa, Tanzania; 4 International Institute of Tropical Agriculture (IITA), C/O World Vegetable Centre, Arusha, Tanzania; International Food Policy Research Institute, UNITED STATES

## Abstract

In this paper, the authors use survey data from over 800 households to examine the impact of demonstration plots and associated activities (distribution of small packs of agricultural inputs) on smallholder farmers’ decisions to buy agricultural inputs in Tanzania. Using propensity score matching and inverse probability-weighted adjustment models, the authors estimated the effect of access to demonstration plots alone and demonstration plots combined with small packs of agricultural inputs on a household’s decision to purchase improved inputs. The results indicate that access to demonstration plots and demonstration plots with small packs increased the probability of purchasing improved inputs by 13–17 percentage points. This paper suggests that demonstration plots and demonstration plots with small packs are an effective model for enhancing improved technology adoption and are further increased when those inputs are available within a 5km radius. The results point to the importance of strengthening farmers’ organizations and last-mile agricultural input suppliers in order to enhance and facilitate access to information, appropriate production techniques, and improved inputs. The results also indicate the importance of investing in infrastructure to reduce transportation costs that limit market efficiency for appropriate technologies.

## 1. Introduction

### 1.1 Background

Despite some progress over the past years, agricultural productivity in sub-Saharan Africa is still low and far below potential [1]. In Tanzania, smallholder agriculture is the main source of livelihoods for most of the population, employing over 70% of the population and contributing 25% to the Gross Domestic Product [2]. The fact that most of the population contribute only 25% of GDP is indicative of the low productivity and therefore high vulnerability to food and income insecurity. The main factors that limit the productivity of smallholder agriculture in the country include land degradation and poor soil fertility; climate variability; crop pests and diseases; low adoption of improved agronomic practices as a result of inadequate access to information and unreliable agro-input supply systems and institutional barriers such as poor markets for inputs and farm products; and poor farmer organization [3].

The Government of Tanzania and its development partners have developed and implemented several policies and programs aimed at improving agricultural productivity and nutrition as summarized by [4]. These include the Tanzania Development Vision (TDV 2025), CAADP country strategy, which translates into the Food Security Development Plan (TAFSIP– 2016/17–2020/21); Agricultural Sector Development Plan II (2016/17–2020/21); the National Multi-Sectoral Nutrition Action Plan (NMNAP– 2016/17–2020/21), District Agricultural Development Plans (DADPs), and the Southern Agricultural Growth Corridor of Tanzania (SAGCOT). Efforts of these plans have realized some results but more still needs to be done. At the global level, in 2010, the United States government launched a hunger and food security initiative, Feed the Future (FtF), which is designed to harmonize regional hunger- and poverty-fighting efforts in countries with chronic food insecurity and insufficient production of staple crops. Tanzania was one of the beneficiary countries as part of this initiative. The FtF initiative was designed to thrive on leveraging of partnerships, innovation and host government leadership [5].

One of the FtF investments in Tanzania was the NAFAKA staple value chain project led by ACDI/VOCA. The first phase of the activity (project) was commissioned in 2011 and ran through 2016 with a goal of sustainably reducing poverty and hunger by improving the productivity and competitiveness of maize and rice value chains that offer job and income opportunities for rural households in Tanzania [6]. A second phase of the NAFAKA project focusing on market systems development (NAFAKA II) was launched in September 2016 and will close activities in October 2021. The NAFAKA project partnered with another FtF initiative, the Africa Research in Sustainable Intensification for the Next Generation (Africa RISING) led by the International Institute of Tropical Agriculture (IITA). This project also had a focus on creating opportunities for smallholder farm households to move out of hunger and poverty through sustainably intensified farming systems that improve food, nutrition, and income security, particularly for women and children, and conserve or enhance the natural resource base.

Given the centrality of agricultural extension and advisory services for addressing rural poverty and food insecurity [7], the two interventions made investments in this component. Particularly, demonstrations plots were used in conjunction with other extension methods and techniques given their role in enabling farmers to learn first-hand about improved technologies [8] and then complemented with small packs of agro-inputs and extension training activities to stimulate farmers’ trial and experimentation before making adoption decisions as suggested by [9]. The objective of this study is therefore to assess whether these influence farmers’ decisions to adopt agro-inputs when compared to farmers in non-project locations.

### 1.2 Study context and related literature

Our study focuses on activities related to maize production in Tanzania, the largest producer in East Africa. Maize is also the main staple crop in Tanzania, in addition to rice which NAFAKA and Africa RISING projects also work with in the country. Both crops are grown by over 90% of farmers in the country. The implementation approach for the NAFAKA and Africa RISING projects involved developing a network of rural-based extension service providers (volunteer and government staff), group and association capacity building and enhancing access to agro-inputs through agro-input supply networks. NAFAKA has additional unique approaches to expanding market and trade and engaging with public and private sectors to play active roles in enhancing smallholder livelihoods across the value chain.

The intervention further focuses on the establishment of demonstration plots for farmer learning and experimentation, thereby providing an opportunity for them to observe the benefits of crop varieties, good agronomic practices (GAPs) and natural resource management. The plots are managed by the village-based extension staff and lead farmers who use them to provide direct training to farmers with technical support from NAFAKA and Africa RISING scientists. Another utility of the demonstration plot model is that it is anticipated to stimulate farmers’ purchase of agro-inputs after observing clear benefits of the technologies at the plots.

Demonstration plots, and later farmer field schools have been a cornerstone of agricultural extension services in Tanzania [8, 10, 11]. Demonstration plots, when well planned, designed and implemented, provide an opportunity for beneficiaries to, among others, see the technologies together with their benefits as well as interact with the scientists, extension staff and other actors in development and research. The beneficiaries are also able to have key questions answered and doubts cleared thereby providing further reinforcement on their decisions to adopt the demonstration technologies. Several studies related to demonstration plots and cereals production have been conducted in East Africa. For instance, [12–14], analyzed the impact of demonstration plots and other factors on farming practices, while [15, 16], and specifically focused on the impact of demonstration plots on cereals farming in East Africa.

Results of these studies show different benefits of demonstration plots on household income and investment. Notably [12], concludes that an extension program featuring demonstration plots contributed to statistically significant increases in household income and investment. Likewise [13], found a highly statistically significant increase in farm income for farmers attending Farmer Training Centers and demonstration plots. In contrast [14], showed that although training programs featuring demonstration plots were linked to adoption decisions, the impact was limited by capital constraints. However, to our knowledge, very few studies explicitly focused on the extent to which demonstration plots, either in isolation or in combination with other activities influenced farmers’ decisions to purchase and use inputs associated with the demonstration technologies. We aim to contribute to the growing literature on agricultural extension by assessing the effect of demonstration plots and demonstration plots combined with small packs of inputs on the purchase of improved agricultural inputs using a unique and recent household-level data. Precisely, we use the propensity score matching (PSM) and the doubly robust inverse probability weighted regression adjustment (IPWRA) models to estimate the average treatment effects. The IPWRA provides efficient estimates by allowing the modelling of both the outcome and the treatment equations and requires that only one of the two models are correctly specified to consistently estimate the impact [17].

The rest of the paper is organized as follows. In the next section, we present the sampling strategy and data collection procedure. Section 3 lays out the empirical framework whereas section 4 presents the results and discussion. The last section draws conclusions and recommendations.

## 2. Materials and methods

### 2.1 Sampling and data collection

**Fig 1:**
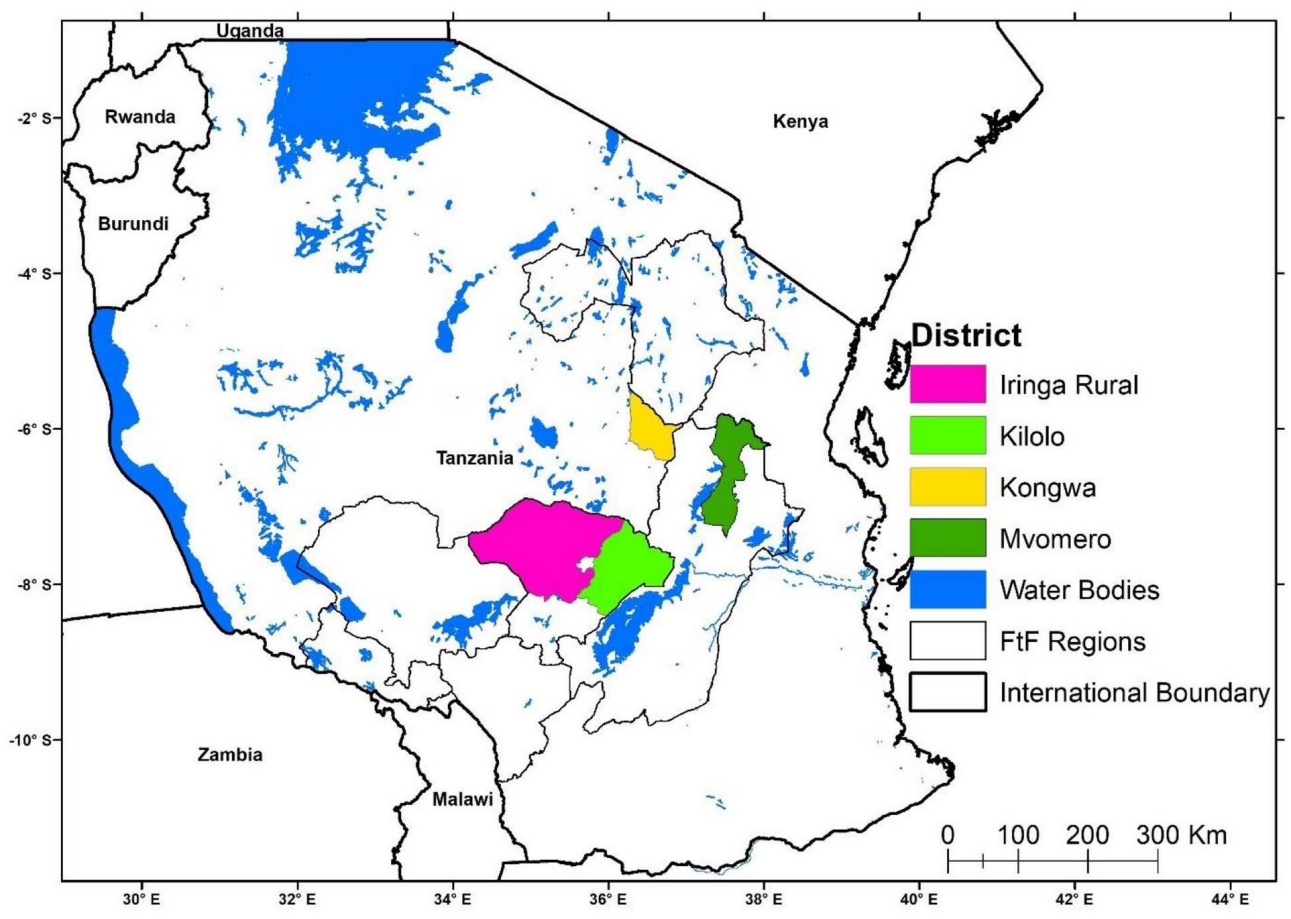
Districts where the study was conducted.

The study was conducted in: (i) districts where NAFAKA/Africa RISING was operational from the inception of the projects in 2012 (i.e. Kongwa and Mvomero districts); (ii) districts where NAFAKA/Africa RISING started operating in 2016 (i.e. Iringa Rural and Kilolo districts). These districts are shown in Fig 1. As the first stage of the sampling procedure, these districts were selected purposively. Specifically, Kongwa and Iringa districts were selected to participate in the Africa RISING/NAFAKA projects because they had some of the most food-insecure villages in Tanzania. There are parts of these districts that are semi-arid with unreliable and unevenly distributed rainfall associated with frequent cycles of drought and flooding pushing agro-pastoral and smallholder farming households over the edge. Without the benefit of modern farming technologies, farmers typically rely on low-yielding practices and crop varieties. Contrary to Kongwa and Iringa, Mvomero and Kilolo districts have stable and reliable rains but the farming communities typically own small land sizes and most are remote and thus far from markets. Also, they face threats of land degradation and diminishing farm outputs although they are using improved seeds and fertilizers.

In the second stage, sets of villages were selected as “treatment” villages and “control” villages from each district. Treatment villages are those where NAFAKA/Africa RISING had interventions and control villages are those that did not receive any project intervention. The control villages were identified in the same agroecological zone as treatment villages. Farmers in these villages rely exclusively on public extension services provided by village agricultural extension officers (VAEOs). The VAEOs operate in a challenging work environment with limited travel and logistical support, limited training on new technologies and minimum supervision. There were no established demonstration plots in the control villages at the time of conducting this study.

VAEOs were engaged by the projects through additional GAP trainings. Each VAEO was also tasked to establish a demonstration plot in collaboration with existing farmer groups in his or her village of operation. Besides working with community members, the treatment villages also benefited from the ACDI/VOCA Village-Based Agricultural Agents (VBAAs). These agents were selected by community members to complement the VAEOs’ work and provide immediate GAP knowledge in the villages after completing a series of training sessions conducted by the projects. In addition to co-managing the demonstration plots, the VBAAs also provided small packs of agro-inputs to farmers and, where supported, established agricultural input shops thereby increasing farmers access to these inputs.

In each of the four NAFAKA/Africa RISING districts, five treatment villages and five control villages were randomly selected for the survey using probability proportional to size sampling (PPS). It is also noteworthy that the study focused on only maize production locations.

Finally based on a sample size calculation considering the total number of NAFAKA/Africa RISING farmers, 400 farmers each were selected from the treatment and control villages to create a total sample size of 800 respondents. However, to account for the non-response rate, more than the minimum target of 20 farmers per village were interviewed in some villages. In total, 866 respondents were interviewed including 444 respondents from treatment villages and 422 respondents from control villages. Nevertheless, due to incomplete data from some of the questionnaires, only 852 households were considered in the analysis.

Data were collected in February 2018 using interviews with respondents from the treatment and control villages. Specifically, a team of well-trained enumerators used an electronic questionnaire on the *Kobo Toolbox* smartphone application to interview the selected survey respondents. The interviews were conducted in the local language (Swahili) to ensure that the questions could be easily understood by all respondents. The use of an electronic questionnaire was very cost-effective and allowed for highly efficient survey enumeration.

### 2.2 Ethics statement

"The data was collected through household surveys and data were analyzed anonymously. The participants in the survey were selected from the beneficiaries and non-beneficiaries of the Africa RISING and NAFAKA project. A clear explanation of the objectives of the survey was given to the participants and all of them were asked for their verbal informed consent to willingly participate in the study. If the respondents declined to be interviewed, the reasons for their refusal were also recorded and no one was forced to participate in the survey."

### 2.3 Conceptual framework and empirical procedure

In this study, we view the decisions of the farmer to visit a demonstration plot in a given period to be derived from the maximization of expected utility subject to cash, credit, and other constraints [18]. In the spirit of other studies in the vein (e.g. [19–21]), let (*U*_*E*_) represent the utility to the farmer from accessing a demonstration plot and let (*U*_*N*_) represent the utility from not visiting a demonstration plot. A farmer will choose to visit a demonstration plot if $D_{i}^{*}=\;U_{E}-U_{N}>0$. $D_{i}^{*}$ is a latent variable determined by observed characteristics (*Z*_*i*_) which include group membership, ownership of household assets, livestock, household head socioeconomic characteristics and average annual rainfall and; the error term (*e*_*i*_) such that:

$$D_{i}^{*}=\beta Z_{i}+e_{i}\;\text{with}\;D_{i}=\{\begin{array}{ll} 1 & if\;D_{i}^{*}>0\\ 0 & otherwise \end{array}$$ (1)

where *D*_*i*_ is a binary indicator variable that equals 1 if a farmer visits a demonstration plot and/or demonstration plot with small packs (hereafter referred to as treated) and zero otherwise (hereafter referred to as not treated) and *β* is a vector of parameters to be estimated.

#### 2.3.1 Propensity score matching

As explained above, we envisage that accessing demonstration plots and demonstration plots with small packs will encourage farmers to invest in improved inputs. To estimate the impact of the demonstration plots and/or demonstration plots with small packs on the agro-input purchase, we used the propensity score matching approach [21–23]. Specifically, we used the Average Treatment Effect on the Treated (ATT) to measure the impact which is the average difference between expected outcome values with and without treatment for those who had access to demonstration plots and/or demonstration plots with small packs. Following [24, 25], the ATT can be defined as:

$$\begin{array}{ll} ATT & =E(Y_{1i}-Y_{0i}|D_{i}=1)\\ & =E(Y_{1i}|D_{i}=1)-E(Y_{0i}|D_{i}=1) \end{array}$$ (2)

Where *E* (.) is the expectation operator, *Y*_1*i*_ is the outcome for the treated households, *Y*_0*i*_ is the counterfactual outcome for the same household and *D*_*i*_ is as defined as above. One problem that arises in estimating Eq (2) is that we can only observe either *Y*_1*i*_ or *Y*_0*i*_ but not both of them for each household. Using the mean outcome of untreated individuals may lead to selection bias because it is most likely that components which determine the treatment decision also determine the outcome variable of interest especially in non-experimental studies [24]. To address this problem, we use PSM. The PSM uses propensity scores to match every individual observation of treated households with an observation with similar characteristics from the non-treated or control group. The propensity score is the conditional probability of assignment to the treatment given a vector of observed covariates [26]. In an ideal situation, random assignment to treatment is the best way of correcting for selection bias because all beneficiaries would have an equal chance of being assigned to each treatment [27]. However, implementing a randomized experiment is quite expensive and was not feasible in our study. Other methods of correcting for selection bias due to both observed and unobserved characteristics such as Instrumental Variable (IV) techniques impose distributional and functional form assumptions, such as linearity on the outcome equation and extrapolating over regions of no common support, where no similar treated and non-treated observations exist [21]. Although PSM does not correct for selection bias due to unobservables, it does not impose distributional assumptions. Incorporating propensity scores in Eq (2) leads to:

$$ATT=E[D_{i}=1,\;p(X_{i})]-E[D_{i}=0,\;p(X_{i})]$$ (3)

Where *p*(*X*_*i*_) are the propensity scores estimated from Eq (1) and defined as:

$$p(X_{i})=\text{Pr}(D_{i}=1|X)=F\{h(X)\}=E(D_{i}|X)$$ (4)

where *X* is a vector of covariates based on observed characteristics (i.e. the same as *Z*_*i*_ in Eq (1)) and *F*{.} is a normal cumulative distribution function. In the estimation of the ATT, we used the nearest neighbour and kernel-based matching algorithms.

PSM estimation relies on two important assumptions; the conditional independence and overlap assumptions. The conditional independence assumption (CIA) states that the treatment assignment is essentially randomized when we condition on a rich set of covariates. It suggests that that systematic differences in outcomes between treated and comparison households with the same values for covariates are attributable to treatment [25]. The CIA assumption cannot be tested and only relies on conditioning on a rich set of observed covariates. The overlap assumption on the other hand states that conditioning on a set of covariates, everyone has a positive probability of receiving treatment (also known as the overlap assumption). We test this assumption in the subsequent sections.

#### 2.3.2 Inverse probability weighted regression adjustment

As a robustness check, we also estimated the ATT using the inverse probability weighted regression adjustment (IPWRA) which is sometimes referred to as a doubly robust estimator [17, 28]. Like propensity score matching (PSM), the IPWRA only accounts for observed and does not control for unobserved heterogeneity. One of the drawbacks of the PSM method is that biased estimates may be obtained if the propensity score model is misspecified [28]. Unlike PSM, the IPWRA method provides efficient estimates by allowing the modelling of both the outcome and the treatment equations and requires that only one of the two models are correctly specified to consistently estimate the impact. It combines the inverse probability weighting (treatment model) with regression adjustment (outcome model) to achieve this. The ATT for the IPWRA can be specified as:

$$\begin{array}{ll} ATT_{IPWRA} & =N^{-1}\overset{N}{\underset{i=1}{\sum }}[(\alpha_{1}^{*}+\beta_{1}^{*}X_{i})-(\alpha_{0}^{*}+\beta_{0}^{*}X_{i})]\\ & =[\left(\alpha_{1}^{*}-\alpha_{0}^{*})+\bar{X}{\;}_{1}(\beta_{1}^{*}-\beta_{0}^{*}\right)] \end{array}$$ (5)

Where $\left(\alpha_{1}^{*},\beta_{1}^{*}\right)$ are attained from the inverse probability-weighted least squares problem for the treated group

$${\text{min}}_{\alpha_{1}\beta_{1}}\sum_{i=1}^{N}\frac{{\left(y_{i}-\alpha_{1}^{*}-\beta_{1}^{*}X_{1}\right)}^{2}}{\hat{p}(X,\hat{\gamma })}$$ (6)

and $\left(\alpha_{0}^{*},\beta_{0}^{*}\right)$ are attained from the inverse probability-weighted least squares problem for non-treated

$${\text{min}}_{\alpha_{0}\beta_{0}}\sum_{i=0}^{N}\frac{{\left(y_{i}-\alpha_{0}^{*}-\beta_{0}^{*}X_{0}\right)}^{2}}{1-\hat{p}(X,\hat{\gamma })}$$ (7)

The * on the estimated parameters *α*, *β*, and *X* describes the double robustness result; $\hat{p}=(X,\hat{\gamma })$ are the estimated propensity scores. Note that the *X*’s are defined as above.

## 3. Results and discussion

### 3.1 Descriptive results

Table 1 shows the outcome and explanatory variables considered in the study, drawn from the extensive literature on agricultural extension (e.g. [7, 29–31]). On average, 33% of the households purchased improved agricultural inputs. The improved inputs include fertilizers, crop protectants, and improved seeds. Accordingly, ‘improved inputs’ as used in this study is a purchase of the combination of improved seeds, fertilizers and crop protectants. A household was considered to have purchased improved inputs if they bought any one or a combination of the inputs. Results in Table 1 also show that about 37% of the households had access to demonstration plots while 33% had access to demonstration plots and received a small pack of improved inputs. Demonstration plots are farmer-owned and farmer-managed plots of land used by village-based extension agents (VBAA), village agricultural extension officers (VAEOS) or Lead Farmers as a platform for training farmers on GAPs. They are designed to facilitate positive changes in farmer practices through the integration of core behaviours in their farm activities such as proper land preparation, proper spacing, use of fertilizer and improved seeds, soil and water management, pest and disease control, and pre-harvest/harvest/post-harvest practices. Such practical training in the demonstration plots is the initial step towards developing knowledge and skills for farmers to build their capacity to adopt improved practices and, in turn, increase marginal sales and yields. Farmers were asked whether they have ever accessed the project demonstrations at least once for purposes of accessing knowledge and skills which they transfer to their farm operations.

**Table 1:** Descriptive statistics and the definition of variables.

Variable	Definition	Mean	SD
*Outcome variable*			
Improved inputs	= 1 if household purchased improved inputs, 0 otherwise	0.33	0.47
*Treatment variables*			
Demonstration plots	= 1 if a household had access to a demonstration plot, 0 otherwise	0.37	0.48
Demonstration plots with small packs	= 1 if a household had access to a demonstration plot and received small packs of agricultural inputs, 0 otherwise	0.33	0.47
*Explanatory variables*			
Household size	= Total household size (number)	5.19	2.19
Household head education	= Household head education (rank)	1.88	1.11
Household head sex	= 1 if household head is male, 0 otherwise	0.22	0.42
Household head youth	= 1 if household head is a youth, 0 otherwise	0.25	0.43
Farm size	= Land owned by households (ha)		
Livestock ownership	= Livestock ownership measured in Tropical Livestock Units (TLU)	2.51	12.23
Wealth index	= Household wealth index	0.00	1.575
Phone ownership	= 1 if household owned a mobile phone, 0 otherwise	0.82	0.38
Bicycle	= 1 if a household uses a bicycle as a means of transport	0.34	0.47
Farmer group	= 1 if a household is a member of a farmer group, 0 otherwise	0.58	0.49
Lending group	= 1 if a household is a member of a lending group, 0 otherwise	0.20	0.4
Tarmac road	= 1 if a household has access to a tarmac road	0.02	0.14
Average rainfall	Average rainfall (mm)	703.8	263.1

In contrast, small packs represent agro-inputs marketing approach designed to remove barriers to smallholder farmers’ adoption of improved seeds and fertilizers in rural and remote areas suffering from the prevalence of expired and counterfeit inputs, particularly seed, leading to low confidence among farmers that improved seeds and fertilizers justify their investment costs. It involves packs of seed or fertilizer ranging from 50 to 250 grams being distributed for free to farmers by VBAAs for them to try out for purposes of eliminating doubt, increasing awareness, and generating interest in purchasing these inputs.

To capture household capital endowments, we include household size, education and wealth. The size of the household is usually a proxy of household labour availability and previous studies have shown that larger households are more likely to adopt improved agricultural technologies [32]. We expect access to demonstration plots to increase with education because generally, education broadens interest in access to information and services, supporting innovation. We proxy for wealth using a wealth index constructed using principal component analysis (PCA). The wealth index includes variables measuring various dwelling characteristics: access to electricity, toilet quality, roof quality, floor quality, and the number of rooms. Besides, mobile phone ownership and livestock ownership are included in our models but are not part of the constructed wealth index. It is expected that wealthier households are more likely to access demonstration plots and use improved agricultural inputs because, in most cases, improved agricultural inputs are expensive.

Social capital is important in not only facilitating access to improved agriculture technologies but also in mitigating against production and net returns risks. We measure social capital in terms of farmer and lender group membership. Group membership indicates the intensity of contacts with other farmers, hence farmers who do not have contacts with extension agents may still be informed about new technologies by their colleagues [33]. Results indicate that about 58% of the sample households were members of a farmer group.

Finally, most countries in sub-Saharan Africa, including Tanzania are subject to environmental problems such as droughts and uneven distribution of rainfall and may also affect the decision to purchase improved agricultural inputs. We capture the variability in rainfall by including a rainfall variable which measures the amount of rainfall that was received in the 2016–17 farming season.

Table 2 shows the descriptive statistics disaggregated by access to demonstration plots. There is a statistically significant difference between the two groups for several variables notably, households which accessed demonstrations had significantly higher means for several variables related to improved inputs, wealth and access to resources.

**Table 2:** Descriptive statistics by access to demonstration plots.

Variables	Accessed demonstration plots	Did not access demonstration plots	Mean difference
*Outcome variable*			
Improved inputs	0.42	0.28	0.13***
*Explanatory variables*			
Household head education	1.94	1.84	0.10
Household head sex	0.77	0.78	- 0.01
Household head youth	0.22	0.27	-0.05*
Household size	5.33	5.11	0.22
Farm size	2.54	2.43	0.11
Livestock ownership	2.92	2.28	0.64
Wealth index	0.15	-0.09	0.24**
Phone ownership	0.87	0.80	0.08***
Bicycle	0.45	0.27	0.17***
Farmer group	0.21	0.07	0.13***
Lending group	0.27	0.16	0.11***
Average rainfall	692.50	710.40	-17.91
Tarmac road	0.041	0.01	0.04***

Note:

* p<0.10,

** p<0.05,

*** p<0. 001.

The difference is measured by the two-sample *t-*test with equal variances.

### 3.2 Empirical results

A logit model was used to estimate the probability of access to demonstration plots and demonstration plots with small packs. Table 3 shows the marginal effects, with standard errors clustered at the village level for the results in columns 2 and 4. Even though the main objective of the study was to examine the impact of extension (i.e. access to demonstration plots and demonstration plots with small packs) on the purchase of improved inputs, we briefly discuss the results in Table 3. The results indicate that female-headed households were 8% and 7% less likely to access demonstration plots and demonstration plots with small packs and these results are in line with the findings of [34]. Consistent with previous studies on extension [e.g. 35], we found that households with larger farms were less likely to access demonstration plots with small packs. This is plausible because most extension agents are more likely to target smallholder farmers. The results also show that access to demonstration plots and demonstration plots with small packs increased with livestock ownership and wealth index. Wealthier households are usually in a better position to bear the possible risks and costs associated with accessing demonstration plots and may have the ability to finance the purchase of inputs. The results also indicate that mobile phones increased the likelihood of accessing demonstration plots by 8%, which is likely because mobile phones are an important information access tool allowing farmers to exchange information regarding the location of the demonstration plots for instance.

**Table 3:** Determinants of demonstration plots and demonstration plots with small packs.

Variables	Demonstration plots		Demonstration plots with small packs	
	With village cluster std errors	Without village cluster std errors	With village cluster std errors	Without village cluster std errors
Household head education	0.01 (0.02)	0.01 (0.01)	0.01 (0.02)	0.01 (0.01)
Household head sex	-0.08* (0.04)	-0.08** (0.04)	-0.07* (0.04)	-0.07* (0.04)
Household head youth	-0.03 (0.04)	-0.03 (0.04)	-0.05 (0.04)	-0.05 (0.04)
Household size	-0.00 (0.01)	-0.00 (0.01)	-0.00 (0.01)	-0.00 (0.01)
Farm size	-0.01 (0.01)	-0.01* (0.01)	-0.02** (0.01)	-0.02** (0.01)
Livestock ownership	0.00** (0.00)	0.00** (0.00)	0.00** (0.00)	0.00** (0.00)
Wealth index	0.03 (0.02)	0.03** (0.01)	0.03* (0.02)	0.03** (0.01)
Phone ownership	0.08* (0.05)	0.08* (0.05)	0.05 (0.04)	0.05 (0.04)
Farmer group	0.25*** (0.06)	0.25*** (0.04)	0.23*** (0.06)	0.23*** (0.04)
Lending group	0.12** (0.05)	0.12*** (0.04)	0.13** (0.05)	0.13*** (0.04)
Average rainfall	0.00** (0.00)	0.00*** (0.00)	0.00** (0.00)	0.00*** (0.00)
Tarmac road	0.39** (0.12)	0.39** (0.13)	0.34*** (0.09)	0.34** (0.11)
Bicycle	0.10** (0.04)	0.10** (0.03)	0.10** (0.04)	0.10** (0.03)
Kilolo district	-0.41** (0.16)	-0.41*** (0.05)	-0.42** (0.16)	-0.42*** (0.05)
Iringa district	-0.00 (0.17)	-0.00 (0.04)	0.02 (0.14)	0.02 (0.04)
Mvomero district	-0.51** (0.20)	-0.51*** (0.07)	-0.46** (0.19)	-0.46*** (0.07)
Observations	852	852	852	852

Note: Standard errors in parentheses.

* p<0.10,

** p<0.05,

*** p<0.001.

Similar to the results found by [36, 37], results in Table 3 indicate that access to demonstration plots and demonstration plots with small packs increased with membership in farmer and lending groups by between 12%–25%. Bicycle ownership and access to a tarred road are proxies for transport equipment and transaction costs associated with accessing information through demonstration plots. Specifically, the results show that the probability of accessing demonstration plots increased by 39% and that of demonstration plots with small packs by 34%. Accessing a tarred road also increased the propensity to access demonstration plots and demonstration plots with small packs by 34%, suggesting that farmers who are located near a tarred road were more are likely to access extension services [37].

Finally, district dummies reflect the agro-ecological and resource differences in the four districts. Relative to Kongwa district, farmers in Kilolo and Mvomero districts were less likely to have access to demonstration plots and demonstration plots with small packs.

### 3.3 PSM estimates of the impact of access to demonstration plots and demonstration plots with small packs on the purchase of improved inputs

**Fig 2:**
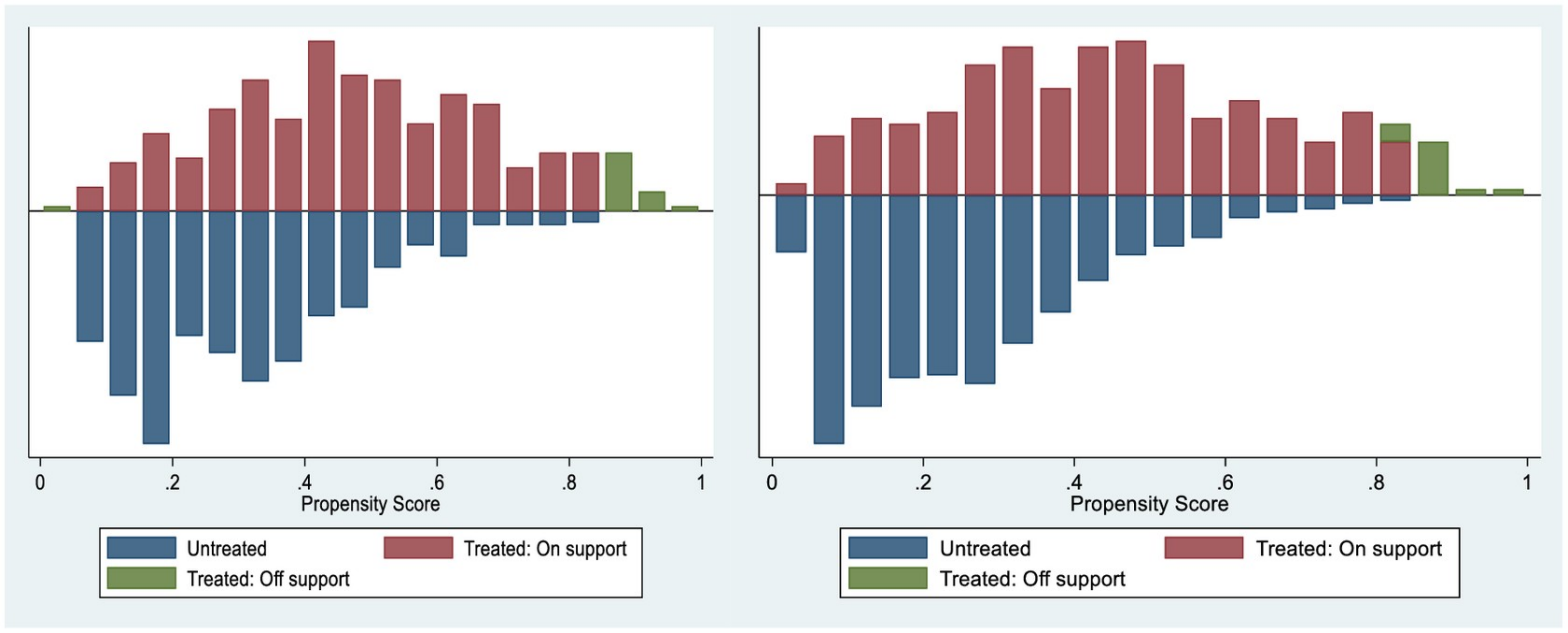
Propensity score distribution and common support for propensity score estimation. Note: ‘‘Treated: on support” indicates the observations in the treated group (demonstration plots and demonstration plots with small packs) have a suitable comparison. ‘‘Treated: off support” indicates the observations in the treated group that do not have a suitable comparison.

The logit model results presented above (with standard errors clustered at village level) were used to generate propensity scores upon which the observed characteristics were balanced across the treated and non-treated households. Before estimating the causal effects of demonstration plots and demonstration plots with small packs on the purchase of improved inputs, we first tested whether the overlap assumption was satisfied and accessed the quality of matching on propensity scores. Fig 2 shows the propensity score distribution and common support for propensity score estimation. The results show that the common support condition is satisfied as there is substantial overlap in the distribution of the propensity scores of the treated and non-treated groups.

Since PSM relies on conditioning on propensity scores and not on all the covariates, it must be checked if the matching procedure can balance the distribution of the relevant variables in the control and treatment groups [25]. Table 4 presents the results from covariate balancing tests before and after matching. The reduction in the mean absolute standardized bias between the matched and unmatched models was used to assess the balancing of covariates. The balancing tests in Table 4 showed a substantial reduction in the mean absolute bias between the matched and unmatched models, with no significant differences after matching. The total bias reduction ranged from 71–76% and this indicates that PSM was successful in reducing selection bias due to observed characteristics.

**Table 4:** Matching quality indicators before and after matching.

Treatment (Matching algorithm)	Pseudo *R*^2^ Before matching	Pseudo *R*^2^ after matching	LR *X*^2^ (*p*-value) Before matching	LR *X*^2^ (*p*-value) After matching	Mean standardized bias before matching	Mean standardized bias after matching	Total% |bias| reduction
Demonstration plots (NNM)	0.14	0.007	160.82 (*p* = 0.00)	6.12 (*p* = 0.96)	17.8	4.70	73.60
Demonstration plots with small packs (NNM)	0.16	0.01	169.86 (*p* = 0.00)	10.14 (*p* = 0.86)	18.5	5.30	71.35
Demonstration plots (KBM)	0.14	0.01	160.82 (*p* = 0.00)	5.43 (*p* = 0.99)	17.80	4.30	75.84
Demonstration plots with small packs (KBM)	0.16	0.01	169.86 (*p* = 0.00)	8.86 (*p* = 0.92)	18.5	5.40	70.81

^1^NNM = three neighbours matching and common support.

^2^KBM = kernel-based matching (Epanechnikov) with bandwidth 0.03 and common support.

The effects of demonstration plots and demonstration plots with small packs on the purchase of improved inputs estimated with the nearest neighbour (NNM) and kernel-based matching (KBM) models are presented in Table 5. The results from the two models are similar (albeit with different treatment effects magnitudes) and they indicate that the probability of purchasing improved inputs increased with access to demonstration plots and demonstration plots with small packs. In the NNM model, visiting a demonstration plot increased the probability of acquiring inputs by 13 percentage points. The households that received small packs in combination with access to demonstration plots were also likely to procure improved inputs by 15 percentage points as compared to the non-treated households (Table 5). The results for the KBM matching algorithm can be interpreted similarly.

**Table 5:** Impact of demonstration plots and demonstration plots with small packs on access to improved agricultural inputs (PSM).

Treatment variable	Matching algorithm	Mean of outcome variables based on matched observations		
		Treated	Non-treated	ATT
Demonstration plots	NNM	0.40	0.27	0.13*** (0.04)
Demonstration plots with small packs	NNM	0.41	0.26	0.15*** (0.05)
Demonstration plots	KBM	0.41	0.27	0.13*** (0.04)
Demonstration plots with small packs	KBM	0.41	0.25	0.16 ** (0.04)

Standard errors in parentheses * p<0.10, ** p<0.05, *** p<0.001.

^1^NNM = three neighbors matching and common support.

^2^KBM = kernel-based matching (Epanechnikov) with band width 0.03 and common support.

### 3.4 Sensitivity analysis and robustness checks

#### 3.4.1 Sensitivity analysis with Rosenbaum bounds

Since the estimation of treatment effects with PSM is based on observed characteristics, a hidden bias may arise if treated and non-treated individuals differ on unobserved variables which simultaneously affect assignment into treatment and the outcome variable. Using the bounding approach suggested by [38], we assess how strongly an unobserved factor may influence the selection process to invalidate the results of PSM analysis [25]. Considering that our outcome variable is binary, we use the Mantel-Haenszel (MH) bound proposed by [39]. The results in Table 6 indicate that the treatment effects were quite robust to the presence of hidden bias at different critical levels of hidden bias (Γ). Across the different treatment variables, the level at which we start to question our conclusion of a positive effect of demonstration plots and demonstration plots with small packs on improved inputs purchase ranges from Γ = 1.4–1.6. This implies individuals differ in their odds of treatment by a factor of 40–60%, in terms of unobserved covariates. These values or bounds reflect “worst-case scenarios” and hence do not indicate the presence of selection bias but only tell us how strong the selection bias should be to invalidate our conclusions [25].

**Table 6:** Rosenbaum bounds for treatments effects of demonstration plots and demonstration plots with small packs on the purchase of improved inputs.

Treatment variable	Gamma (Γ)	Q_mh+	Q_mh-	p_mh+	p_mh-
Demonstration plots	1	3.27	3.27	0.00	0.00
	1.2	2.20	4.35	0.01	0.00
	1.4	1.30	5.27	0.10	0.00
	1.6	0.52	6.08	0.30	0.00
	1.8	-0.01	6.80	0.50	0.00
	2	0.61	7.45	0.27	0.00
	2.2	1.16	8.05	0.12	0.00
	2.4	1.67	8.60	0.05	0.00
	2.6	2.14	9.11	0.02	0.00
	2.8	2.57	9.59	0.01	0.00
	3	2.98	10.04	0.00	0.00
Demonstration plots with small packs	1	3.54	3.54	0.00	0.00
	1.2	2.50	4.59	0.01	0.00
	1.4	1.62	5.49	0.05	0.00
	1.6	0.87	6.27	0.19	0.00
	1.8	0.20	6.98	0.42	0.00
	2	0.21	7.61	0.42	0.00
	2.2	0.75	8.20	0.23	0.00
	2.4	1.24	8.74	0.11	0.00
	2.6	1.69	9.24	0.05	0.00
	2.8	2.11	9.71	0.02	0.00
	3	2.51	10.15	0.01	0.00

Notes: N = 852. Gamma is the log odds differential assignment due to unobserved factors. The upper (Q_mh+) and lower (Q_mh-) bounds are Mantel-Haenszel point estimates and; p_mh+ and p_mh- are the significance levels for the upper and lower bounds point estimates.

#### 3.4.2 IPWRA estimates of the impact of access to demonstration plots and demonstration plots with small packs on the purchase of improved inputs

As a key robustness check for the PSM results, we also estimated the IPWRA model and the results are presented in Table 7. The first and second stage results from the IPWRA are presented in Table A1 in S1 Appendix. The first stage results (treatment equation) shows the determinants of access to demonstration plots and demonstration plots with small packs and are like those presented in Table 3. Since our interest was mainly to compare the impact results with those of the PSM, we are not going to interpret the results in Table A1 in S1 Appendix. When estimating the IPWRA model, we also conducted an overidentification test for covariate balance to check whether the covariates were balanced after propensity score reweighting. The results in Table A1 in S1 Appendix indicate that we cannot reject the null hypothesis that the covariates are balanced, implying that there is no evidence that the covariates used remain imbalanced after propensity score reweighting.

**Table 7:** Impact of demonstration plots and demonstration plots with small packs on access to improved agricultural inputs (IPWRA).

Treatment variable	Mean of outcome variables based on weighted observations		ATT
	Treated	Non-treated	
Demonstration plots	0.42	0.26	0.16** (0.05)
Demonstration plots with small packs	0.43	0.26	0.17*** (0.05)

Note: Village cluster robust standard errors in parentheses ** p<0.05 *** p<0.001.

The results show that participating in demonstration increases the probability of households to purchase improved inputs by 16 percentage points. Similarly, the probability of buying improved inputs increased by 17 percentage points for the households who accessed demonstration plots with small packs. The IPWRA results are very similar to the PSM results which gives credence to our PSM results. The results also suggest that our propensity score model was not misspecified.

## 4. Conclusions and recommendations

This article examines the impact of demonstration plots on the use of improved agricultural inputs in Tanzania. Specifically, we use survey data from more than 800 households and a combination of propensity score matching and the doubly robust inverse probability weighted regression models to achieve our objective.

The results indicate that livestock ownership, membership in farmer’s and lending groups, and access to a tarred road were some of the important determinants of access to demonstration plots and demonstration plots with small packs. Overall, the empirical results across our estimation methods used in this study were largely consistent and show increases in input purchase by between 13 percentage points (for demonstration plots) and 17 percentage points (for the combination of demonstration plots with small packs).

The result suggests that strengthening farmers’ organizations and associations are critical for potentially enhancing, not only access to and use of agro-inputs, but also facilitating access to output markets through improved quality, access to information and knowledge as well as facilitating engagement with policymakers [40, 41].

Though both the control and treatment villages had village agriculture extension officers, the results from this study revealed that farmers in treatment villages were more likely to buy improved agricultural inputs, which is the objective of most of the agricultural extension models. The results point to the need for policies to expand past demonstration plots and encourage financial investment to adopt the VBAAs, and farmer organizations models to act as agents for multiple seeds, fertilizers and crop protection companies. Policies that encourage individual entrepreneurs and farmer organizations that can “certify” themselves through VAEOs or the Tanzanian Ministry of Agriculture to act as village agents providing credible GAP knowledge as they identify marketing opportunities will further increase revenues at the village level. These certifications should also be provided with an incubation period that allows new agro-input businesses to increase their cash flow, allowing for an expansion of growth and to establish a customer base.

Furthermore, it is apparent from the results of this study that to enhance smallholder access to demonstration plots, investing in the rural road infrastructure is important. This is because roads not only facilitate access to demonstration plots but also reduce the cost of transportation to the input and output markets.

## Supporting information

S1 Appendix (DOCX)

S1 Data (CSV)
