# Supplementary material for: The impact of demonstration plots on improved agricultural input purchase in Tanzania: Implications for policy and practice
Source: PLoS One. 2021 Jan 15;16(1):e0243896. doi: 10.1371/journal.pone.0243896 (PMC7810322; doi:10.1371/journal.pone.0243896)
Supplement: S1 Appendix — (DOCX) [file pone.0243896.s001.docx]

**S1 Appendix**

Table A1: IPWRA results for determinants and impact of access to demonstration plots and small packs on the purchase of improved inputs

|  |  | Improved inputs | |  |  | Improved inputs | |
| --- | --- | --- | --- | --- | --- | --- | --- |
| Variable | Treatment | Accessed demonstration plots | Did not access demonstration plots |  | Treatment | Accessed demonstration plots and small pack | Did not access demonstration plots and small pack |
| Household head education | 0.06 (0.08) | 0.02 (0.02) | 0.02 (0.02) |  | 0.08 (0.09) | 0.02 (0.02) | 0.03 (0.02) |
| Household head sex | -0.43* (0.23) | -0.00 (0.06) | -0.00 (0.07) |  | -0.41* (0.24) | 0.03 (0.06) | -0.04 (0.07) |
| Household head youth | -0.18 (0.19) | 0.14* (0.07) | -0.12** (0.05) |  | -0.26 (0.20) | 0.12* (0.07) | -0.09* (0.05) |
| Household size | -0.02 (0.04) | 0.02* (0.01) | 0.01 (0.01) |  | -0.01 (0.05) | 0.02* (0.01) | 0.01 (0.02) |
| Farm size | -0.06 (0.04) | -0.01 (0.01) | 0.01 (0.01) |  | -0.09* (0.05) | -0.01 (0.01) | 0.02 (0.01) |
| Livestock ownership | 0.01* (0.01) | 0.00** (0.00) | 0.00 (0.00) |  | 0.02** (0.01) | 0.00** (0.00) | -0.00 (0.00) |
| Wealth index | 0.14 (0.10) | 0.03* (0.02) | 0.04 (0.03) |  | 0.17* (0.09) | 0.03** (0.01) | 0.04 (0.03) |
| Phone ownership | 0.44* (0.24) | 0.10** (0.05) | 0.12 (0.08) |  | 0.26 (0.25) | 0.10** (0.05) | 0.10 (0.09) |
| Farmer group | 1.30*** (0.30) | 0.16 (0.11) | 0.16* (0.08) |  | 1.28*** (0.33) | 0.13 (0.09) | 0.14 (0.09) |
| Lending group | 0.65** (0.25) | 0.11** (0.05) | 0.10 (0.06) |  | 0.73** (0.26) | 0.12** (0.06) | 0.10 (0.06) |
| Average rainfall | 0.00* (0.00) | -0.00** (0.00) | -0.00 (0.00) |  | 0.00** (0.00) | -0.00* (0.00) | -0.00 (0.00) |
| Tarmac road | 2.05** (0.64) | 0.09 (0.13) | 0.25** (0.10) |  | 1.89*** (0.53) | 0.11 (0.14) | 0.25** (0.10) |
| Bicycle | 0.52** (0.23) | 0.01 (0.08) | 0.02 (0.08) |  | 0.58** (0.22) | 0.02 (0.08) | 0.05 (0.09) |
| Kilolo district | -2.13** (0.92) | 0.57*** (0.11) | 0.35* (0.21) |  | -2.35** (0.96) | 0.54*** (0.12) | 0.38* (0.21) |
| Iringa district | -0.02 (0.86) | 0.16** (0.07) | -0.01 (0.15) |  | 0.11 (0.79) | 0.18** (0.07) | -0.03 (0.15) |
| Mvomero district | -2.69** (1.12) | 0.22 (0.16) | 0.01 (0.23) |  | -2.62** (1.17) | 0.17 (0.18) | 0.01 (0.22) |
| Constant | -1.66 (1.13) | 0.12 (0.12) | 0.29 (0.30) |  | -1.95* (1.07) | 0.02 (0.15) | 0.37 (0.32) |
| Overidentification test for covariate balance  *H_0_*: Covariates are balanced | *χ*^2^ (17) = 20.41; *Prob* > *χ*^2^ = 0.2538 | | | | | | |
| Observations | 852 | 317 | 535 |  | 852 | 279 | 573 |

Note: Village cluster robust standard errors in parentheses. * p<0.10, ** p<0.05, *** p<0.001
